# Gray matter changes related to microglial activation in Alzheimer's disease

**DOI:** 10.1016/j.neurobiolaging.2020.06.010

**Published:** 2020-10

**Authors:** Nicolas Nicastro, Maura Malpetti, Elijah Mak, Guy B. Williams, W. Richard Bevan-Jones, Stephen F. Carter, Luca Passamonti, Tim D. Fryer, Young T. Hong, Franklin I. Aigbirhio, James B. Rowe, John T. O'Brien

**Affiliations:** a Department of Psychiatry, University of Cambridge, Cambridge, UK; b Department of Clinical Neurosciences, Geneva University Hospitals, Switzerland; c Department of Clinical Neurosciences, University of Cambridge, Cambridge, UK; d Wolfson Brain Imaging Centre, Cognition University of Cambridge, Cambridge, UK; e Consiglio Nazionale delle Ricerche (CNR), Istituto di Bioimmagini e Fisiologia Molecolare (IBFM), Milano, Italy; f Medical Research Council Cognition and Brain Sciences Unit, Cambridge, UK

**Keywords:** Alzheimer's disease, Neuroinflammation, PET, Grey matter, Atrophy, Cortical thickness, MRI

## Abstract

Neuroinflammation is increasingly recognized as playing a key pathogenetic role in Alzheimer's disease (AD). We examined the relationship between in vivo neuroinflammation and gray matter (GM) changes. Twenty-eight subjects with clinically probable AD (n = 14) and amyloid-positive mild cognitive impairment (n = 14) (age 71.9 ± 8.4 years, 46% female) and 24 healthy controls underwent structural 3T brain MRI. AD/mild cognitive impairment participants exhibited GM atrophy and cortical thinning in AD-related temporoparietal regions (false discovery rate–corrected *p* < 0.05). Patients also showed increased microglial activation in temporal cortices. Higher ^11^C-PK11195 binding in these regions was associated with reduced volume and cortical thickness in parietal, occipital, and cingulate areas (false discovery rate *p* < 0.05). Hippocampal GM atrophy and parahippocampal cortical thinning were related to worse cognition (*p* < 0.05), but these effects were not mediated by microglial activation. This study demonstrates an association between in vivo microglial activation and markers of GM damage in AD, positioning neuroinflammation as a potential target for immunotherapeutic strategies.

## Highlights

• Neuroinflammation is a key player in Alzheimer's disease pathophysiology.
• Microglial activation with ^11^C-PK11195 PET was assessed in patients with Alzheimer's disease.
• Increased microglial activation was observed in temporal cortices.
• Microglial activation was related to parieto-occipital atrophy and cortical thinning.
• It did not mediate the effects of structural damage on cognitive decline.

## 1 Introduction

Alzheimer's disease (AD) causes structural brain abnormalities in the gray matter (GM) volume and cortical thickness, especially in temporoparietal regions (Du et al., 2007; Karas et al., 2003; Richards et al., 2009). There is also evidence that brain inflammation plays a key role in the etiopathogenic cascade of AD (Edison et al., 2008). One aspect of neuroinflammation is microglial activation, which can be measured in vivo with PET ligands such as ^11^C-PK11195 that binds to mitochondrial translocator protein (TSPO), which is overexpressed in activated microglia (Stefaniak and O'Brien, 2016). Increased microglial activation in the medial temporal lobe and precuneus was observed in AD and in its prodromal state of mild cognitive impairment (MCI) (Fan et al., 2015b; Passamonti et al., 2018). Using ^11^C-PK11195, Fan et al. also showed neuroinflammation in MCI. Microglial activation has been shown to decrease longitudinally over a 14-month follow-up in MCI (Fan et al., 2017), although inflammation can rise in progressing AD (Fan et al., 2015b). A separate cross-sectional multimodal MRI and TSPO PET imaging study revealed that higher microglial activation can be associated with relatively preserved GM and hippocampal volume in MCI (Femminella et al., 2019). However, microglial activation negatively correlated with hippocampal volume and brain metabolism in AD (Femminella et al., 2016). These studies highlight the complex temporal relationship between neuroinflammation and neurodegeneration in AD and mixed results call for a replication study. A novel study with a larger sample size, spanning a wider spectrum of disease severity, from amyloid-positive (Aβ+) amnestic MCI to clinically probable AD dementia was therefore conducted. This study examined the relationship between microglial activation, cortical thickness, and GM volume. To achieve this, T1-weighted MRI was analyzed using both volume-based and surface-based morphometry (VBM and SBM, respectively) in relation to ^11^C-PK11195 binding in a cross-sectional cohort of clinically probable AD patients and Aβ+ MCI patients. Our hypothesis was that increased ^11^C-PK11195 binding would correlate with cortical thinning and brain atrophy in typical AD-related regions such as the temporal and parietal cortices. Such negative correlations would support the rationale for anti-inflammatory therapies that can prevent and/or modify the course of AD, especially in terms of reducing the deleterious consequences of inflammation on brain atrophy.

## 2 Materials and methods

### 2.1 Participants

The present work is part of the Neuroimaging of Inflammation in MemoRy and Other Disorders (NIMROD) study (Bevan-Jones et al., 2017). All participants were older than 50 years and had sufficient proficiency in English for cognitive testing. We included 14 participants with probable AD according to McKhann's criteria (McKhann et al., 2011) and 14 patients with MCI defined by National Institute of Aging–Alzheimer's Association criteria (Petersen et al., 2013): a memory impairment at least 1.5 standard deviation below that expected for age and education, the absence of dementia. and a Mini-Mental State Examination (MMSE) score >24/30. In addition, in vivo evidence of amyloid pathology (positive Pittsburgh compound B [PiB] PET imaging) was confirmed in patients with MCI. AD and MCI patients were combined on the basis that these 2 subgroups represent a clinical continuum of the same neuropathologic spectrum.

We also included 24 similarly aged healthy controls, with MMSE scores greater than 26/30, absence of memory complaints, and no unstable or significant medical illness. A detailed clinical and neuropsychological assessment was performed, including the revised Addenbrooke's Cognitive Examination (ACE-R), a 100-point test incorporating 5 key cognitive domains (orientation/attention, memory, verbal fluency, language, and visuospatial) (Mioshi et al., 2006).

Patients were identified from the Memory clinic at the Cambridge University Hospitals NHS Trust, other local memory clinics, and from the Dementias and Neurodegenerative Diseases Research Network (DeNDRoN) volunteer registers. Healthy controls were recruited via DeNDRoN as well as from spouses and partners of participants. Informed written consent was obtained in accordance with the Declaration of Helsinki. The study received a favorable opinion from the East of England Ethics Committee (Cambridge Central Research, Ref. 13/EE/0104).

### 2.2 MRI and PET acquisition and processing

All participants underwent 3T MRI including high-resolution T1-weighted magnetization-prepared rapid gradient echo sequence. GM volume and cortical thickness were assessed using Computational Anatomy Toolbox 12 (CAT12, Structural Brain Imaging Group, University of Jena, Germany) running in MATLAB R2018b version 9.5 (MathWorks Inc., Sherborn, MA, USA). CAT12 performs VBM by providing the voxel-wise estimation of the local amount or volume of a specific tissue compartment. It is based on an automated pipeline including spatial registration to a reference brain, tissue segmentation into GM, white matter and cerebrospinal fluid (CSF), and bias correction of intensity nonuniformities. Subsequent smoothing was performed using an 8mm full width at half maximum as recommended. CAT12 can additionally perform SBM with estimation of cortical thickness and central surface of both hemispheres based on the projection-based thickness method (Dahnke et al., 2013). Using a tissue segmentation to estimate the white matter distance, it projects the local maxima (equaling the cortical thickness) to other GM voxels by using a neighbor relationship described by the white matter distance. Projection-based thickness allows the handling of partial volume information, sulcal blurring, and sulcal asymmetries without explicit sulcus reconstruction. Topological correction, spherical mapping and spherical registration are performed to obtain vertex-wise cortical thickness. Surface maps were smoothed using a 15mm full width at half maximum.

In addition to high-resolution MRI, all AD/MCI patients (n = 28) and 16/24 controls also had ^11^C-PK11195 PET imaging on either a GE Advance PET or a GE Discovery 690 PET/CT scanner. The ^11^C-PK11195 PET image series were aligned across the frames to correct for head motion during data acquisition with Statistical Parametric Mapping 12. The realigned dynamic frames were coregistered to the anatomical images. Before kinetic modeling, regional time-activity curves were corrected for CSF contamination using the two-compartment (brain, CSF) Meltzer method (Meltzer et al., 1999). Binding in each ROI was quantified using nondisplaceable binding potential (BP_ND_) determined with a simplified reference tissue model incorporating vascular binding correction and reference region time-activity curve estimation from supervised cluster analysis using 4 kinetic classes (Yaqub et al., 2012).

Finally, all MCI had ^11^C-PiB PET scan and all were Aβ+ (mean neocortical standardized uptake value ratio > 1.5) with superior cerebellar GM as the reference region (Jagust et al., 2010).

### 2.3 Statistical analyses

Demographic data were analyzed with Stata software version 14.2 (College Station, TX, USA). Assessment of distribution for continuous variables was performed with the Shapiro-Wilk test and visualization of histogram plots, followed by *t*-test or Mann-Whitney U, accordingly. Categorical variables were compared with χ^2^ test. Statistical significance was considered when *p* < 0.05. Correlation between cognitive impairment (according to ACE-R) and regional MRI and PET imaging were performed using linear regressions adjusting for age and total intracranial volume (TIV) (for VBM only).

Average (mean of left and right) individual ROI ^11^C-PK11195 BP_ND_ values based on the Hammersmith n30r83 atlas (www.brain-development.org) were used in a repeated-measures general linear model to test for the main effect of group, ROI, and their interaction, including age and sex as covariates. We derived a composite microglial activation score by averaging the BP_ND_ values of each of the regions showing a significant increase in the AD/MCI group compared with controls (*p* < 0.05).

SBM/VBM group comparisons were performed with nonparametric permutation tests (n = 5′000) and threshold-free cluster enhancement with age and sex as covariates (in addition to TIV for VBM) using the threshold-free cluster enhancement toolbox running in Statistical Parametric Mapping 12. Significance threshold was set at false discovery rate (FDR) *p* < 0.05.

Cross-modal correlation analyses between ^11^C-PK11195 and structural imaging were performed for the AD/MCI group (n = 28) using VBM/SBM maps for multiple regression analyses with the composite ^11^C-PK11195 BP_ND_ score as the independent variable (both partial volume corrected and uncorrected). The following covariates were used: age, sex, TIV (for VBM only), disease severity (measured with ACE-R score), and time interval between ^11^C-PK11195 PET and MRI acquisition. The same analyses were performed without accounting for disease severity (ACE-R score). We also used an FDR-corrected *p* < 0.05 significance threshold adjusting for multiple comparisons (^11^C-PK11195 composite score being correlated with VBM and SBM data). As both brain volume atrophy and preservation have been previously associated with increased microglial activation for AD/MCI (Femminella et al., 2016, 2019), correlational analyses using both contrasts were performed, that is, how microglial activation relates to increased or decreased brain volume and cortical thickness. Mediation analysis was performed to account for direct/indirect/total effects of GM atrophy and cortical thinning on cognition (ACE-R) using the ROI composite ^11^C-PK11195 score as a mediator. We used the hippocampus for VBM and the parahippocampal gyrus for SBM analyses, considering that these regions are classically described as showing GM atrophy and cortical thinning in relation to memory impairment described in AD and MCI. Age and sex were used as covariates.

## 3 Results

### 3.1 Demographics

The demographics and clinical characteristics are reported in Table 1. Both groups were comparable in terms of age, sex distribution, and years of education. As expected, MMSE and ACE-R scores were significantly lower in the AD/MCI group relative to the healthy controls (*p* < 0.0001, Mann-Whitney U test). Scan interval between structural MRI and ^11^C-PK11195 PET was similar among groups (5.2 ± 8.5 months for AD/MCI and 3.8 ± 3.2 months for controls, *p* = 0.31, Mann-Whitney U test).

**Table 1:** Baseline characteristics of included patients

	Controls (n = 24)	AD/MCI (n = 28)	*p* value
Age	70.3 ± 5.9 (59–84)	71.9 ± 8.4 (53–86)	0.47^a^
Female participants	45.8% (11/24)	46.4% (13/28)	0.97^b^
Education	14.2 ± 2.9 (10–19)	13.0 ± 3.1 (10–19)	0.17^c^
MMSE	29.0 ± 1.0 (27–30)	25.3 ± 2.6 (18–30)	<0.0001^c^
ACE-R	93.5 ± 5.0 (79–100)	77.8 ± 9.3 (51–91)	<0.0001^c^

Baseline characteristics of included subjects. Values are mean ± standard deviation (SD) (range).

**Key:** ACE-R, Addenbrooke's Cognitive Examination–Revised; MMSE, Mini-Mental State Examination.

a t-test.

b χ^2^ test.

c Mann-Whitney U test.

### 3.2 ^11^C-PK11195 PET imaging group comparisons

A general linear model of individual ^11^C-PK11195 BP_ND_ showed a significant main effect of group (F_2,43_ = 5.7, *p* < 0.02), main effect of ROI (F_2,24_ = 4.5, *p* < 0.0001), and a group × ROI interaction (F_2,48_ = 2.6, *p* < 0.0001). The group and interaction effects were driven by higher ^11^C-PK11195 BP_ND_ for the AD/MCI group compared with controls in the hippocampus, amygdala, anterior medial temporal, parahippocampal, middle temporal, fusiform, and inferior frontal gyri (all *p* < 0.05). These findings are similar to those described in our previous work in which n = 16 AD/MCI patients overlapped with those included in this study (Surendranathan et al., 2018). A volume-adjusted composite frontotemporal ROI based on these regions was created and ^11^C-PK11195 BP_ND_ was extracted and then used for correlation with VBM/SBM maps in the AD/MCI group.

### 3.3 VBM/SBM MRI imaging group comparisons

**Fig. 1:**
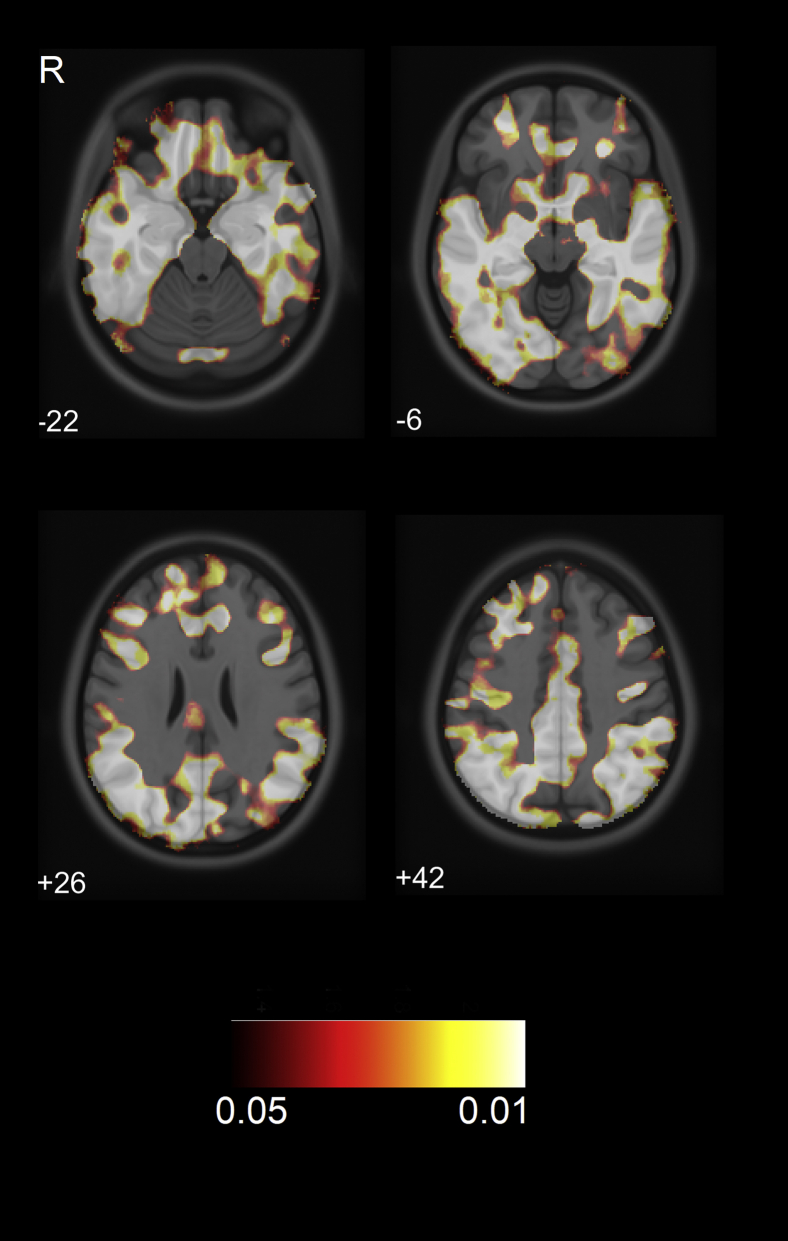
VBM group comparisons showing decreased GM volume (yellow-red) in the AD/MCI group compared to controls (FDR *p* < 0.05). Number corresponds to the Z-axis coordinates in MNI space. R = right. Abbreviations: AD, Alzheimer's disease; FDR, false discovery rate; MCI, mild cognitive impairment; MNI, Montreal Neurological Institute; VBM, volume-based morphometry; GM, gray matter. (For interpretation of the references to color in this figure legend, the reader is referred to the Web version of this article.)

**Fig. 2:**
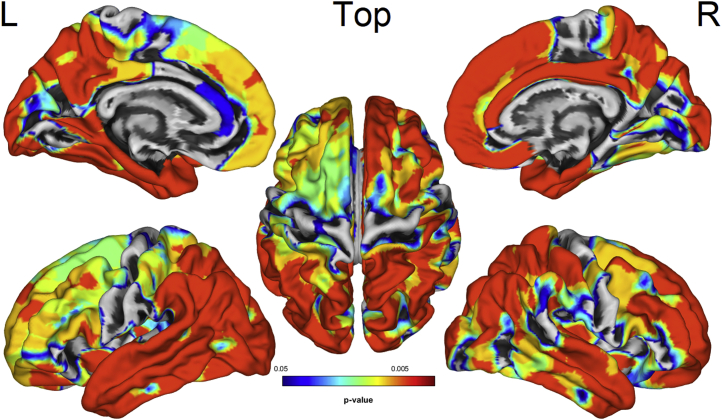
SBM group comparisons showing decreased cortical thickness (blue-red) in the AD/MCI group compared to controls (FDR *p* < 0.05). Abbreviations: AD, Alzheimer's disease; FDR, false discovery rate; MCI, mild cognitive impairment; SBM, surface-based morphometry; L/R, left/right. (For interpretation of the references to color in this figure legend, the reader is referred to the Web version of this article.)

**Supplementary Fig. 1:**
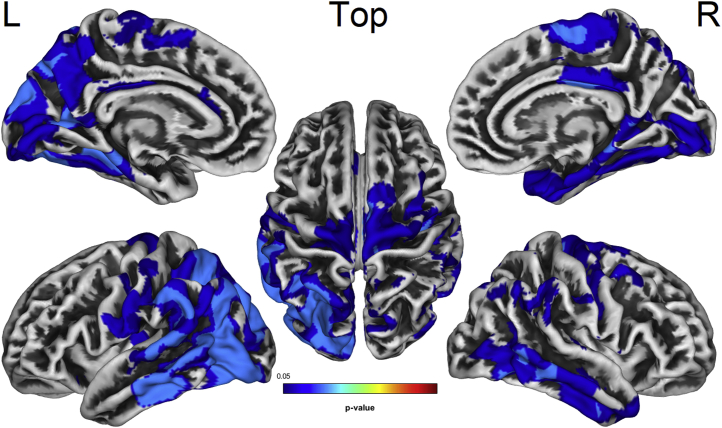
SBM group comparison showing cortical thinning in probable AD (n = 14) compared with MCI subjects (n = 14) (blue-to-red) (FDR-corrected *p* < 0.05).

Voxel-wise VBM group comparisons revealed extensive GM volume reduction for the AD/MCI group relative to controls in frontal, temporal, parietal, and occipital cortices (Fig. 1, FDR-corrected *p* < 0.05). Vertex-wise SBM comparisons showed that AD/MCI exhibited decreased cortical thickness in similar regions, notably in bilateral superior frontal, medial, and lateral temporal, and parietal gyri, as well as precuneus (Fig. 2, FDR-corrected *p* < 0.05). Direct group comparisons between AD (n = 14) and MCI (n = 14) revealed decreased GM volume and cortical thickness for the AD group in lateral temporal, parietal, and occipital areas (Supplementary Fig. 1**,** FDR-corrected *p* < 0.05)**.**

### 3.4 VBM/SBM correlates of ^11^C-PK11195 PET imaging

**Fig. 3:**
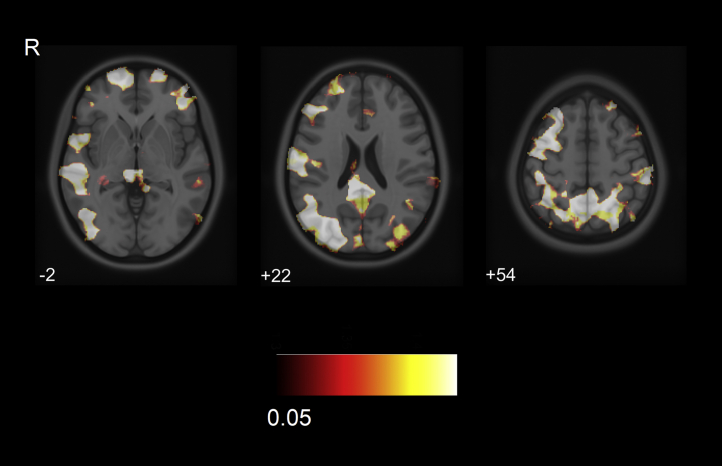
VBM correlation showing gray matter atrophy in frontal, lateral temporal, parietal, and posterior cingulate regions related to higher ^11^C-PK11195 binding in the AD/MCI group (FDR *p* < 0.05). Abbreviations: R, right; AD, Alzheimer's disease; FDR, false discovery rate; MCI, mild cognitive impairment; VBM, volume-based morphometry.

In relation to higher ^11^C-PK11195 binding, AD/MCI patients exhibited a decreased GM volume in several large clusters including bilateral middle frontal, posterolateral temporal, superior parietal, and posterior cingulate regions (FDR *p* < 0.05) (Fig. 3).

**Fig. 4:**
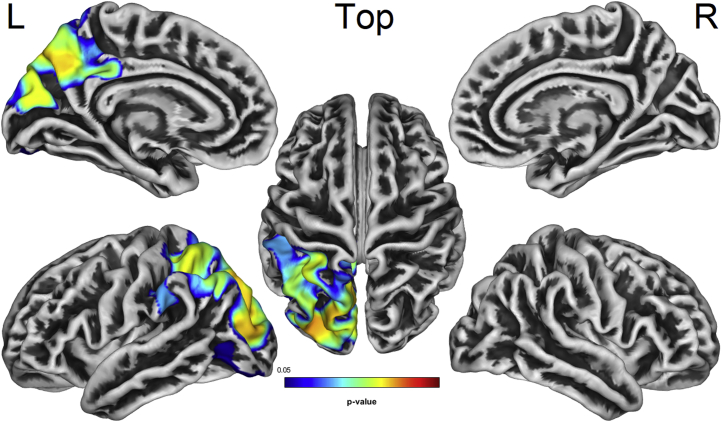
SBM correlation showing cortical thinning in parieto-occipital and cingulate regions related to higher ^11^C-PK11195 binding in the AD/MCI group (FDR *p* < 0.05). Abbreviations: AD, Alzheimer's disease; FDR, false discovery rate; MCI, mild cognitive impairment; SBM, surface-based morphometry; L/R, left/right.

Moreover, increased microglial activation was associated with decreased cortical thickness in the left supramarginal, superior and inferior parietal, precuneus, cuneus, and lateral occipital cortices (FDR *p* < 0.05) (Fig. 4). No significant clusters of GM atrophy or cortical thinning were observed in relation to decreased ^11^C-PK11195 binding. Similar results were obtained when disease severity (ACE-R score) was not used as a covariate, as well as when using nonpartial volume corrected ^11^C-PK11195 binding for the composite frontotemporal ROI. Finally, using a single ROI (the hippocampal gyrus) resulted in similar findings for both VBM and SBM correlational analyses.

### 3.5 Cognitive correlate of MRI and ^11^C-PK11195 PET imaging

**Fig. 5:**
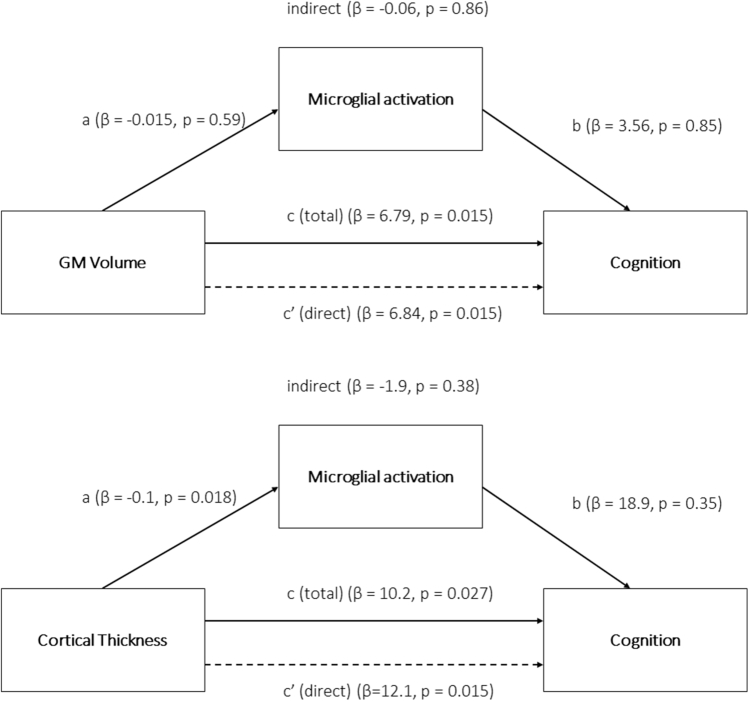
Mediation analysis in the AD/MCI group using hippocampal GM volume (*top*) and parahippocampal cortical thickness (*bottom*) as the independent variable, ^11^C-PK11195 binding as the mediator, and cognition (ACE-R score) as the dependent variable. Covariates include age and sex. Direct, indirect, and total effects of structural damage on cognition are shown. Abbreviations: ACE-R, Addenbrooke's Cognitive Examination–Revised; AD, Alzheimer's disease; FDR, false discovery rate; MCI, mild cognitive impairment; GM, gray matter.

Cognitive impairment (ACE-R total score) correlated with decreased cortical thickness in left parahippocampal gyrus (rho = 0.40, *p* = 0.035) and there was a trend association with decreased bilateral hippocampal GM volume (rho = 0.35, *p* = 0.07) when using the Spearman partial correlations adjusting for age and sex (and TIV for GM volume). No significant correlation between the ACE-R and ^11^C-PK11195 composite binding was observed (Spearman rho −0.23, *p* = 0.23). Mediation analysis showed that both hippocampal GM atrophy and parahippocampal cortical thinning had a direct and total effect on cognition (all *p* < 0.05), whereas no significant effect of ^11^C-PK11195 on cognitive decline was observed (all *p* > 0.34), neither a direct nor an indirect effect mediating GM atrophy or cortical thinning (all *p* > 0.37) (Fig. 5).

## 4 Discussion

In the present study, we show that microglial activation, measured with in vivo ^11^C-PK11195 PET imaging, is associated with frontal, lateral temporal, and parietal GM atrophy, as well as with parieto-occipital cortical thinning. Our AD group showed a typical pattern of GM volume loss and cortical thinning encompassing the whole cortex, especially frontal, temporal, and parietal regions (Du et al., 2007; Karas et al., 2003). In addition, evidence of increased microglial activation was observed in the medial and lateral temporal cortices, that is, the regions known to be most vulnerable to neurodegeneration (Cagnin et al., 2001; Fan et al., 2015b; Passamonti et al., 2018, 2019).

Our findings are based on a cross-sectional analysis accounting for disease severity (measured with the ACE-R scale) and suggest that microglial activation is related to structural impairment in the AD spectrum. Several reports have shown that microglial activation is positively associated with amyloid burden (Fan et al., 2015b) and inversely correlated with glucose metabolism and cognition in AD (Fan et al., 2015a; Femminella et al., 2016). A pathophysiological link between microglial activation and tau deposition has been recently investigated. Using ^18^F-AV-1451 and ^11^C-PK11195 PET, Dani et al. (2018) observed an association between temporal tau retention and microglial activation in AD. Terada et al. (2019) also showed a positive relationship between parahippocampal ^11^C-PBB3 and ^11^C-DPA-713. By contrast, Parbo et al. (2018) did not observe a significant association between tau retention and microglial activation in patients with MCI.

Our findings that microglial activation is related to reduced GM and cortical thickness are apparently contrasting with previous studies reporting that increased TSPO is related to hippocampal volume preservation in MCI (Femminella et al., 2019) as well as to higher cognitive score in AD (Hamelin et al., 2016). Increased microglial activation was also associated with preserved cognition and diffusion tensor imaging metrics in dementia with Lewy bodies (Nicastro et al., 2020; Surendranathan et al., 2018).

However, several caveats need to be considered. First, the relationship between neuroinflammation and degeneration in the AD spectrum has been proposed to be polyphasic, with evidence of an early inflammation peak at MCI onset and further reduction at follow-up (Fan et al., 2015b) while structural integrity of the brain inexorably declines. This would imply that in earlier MCI cases, a relative preservation of both cognition and GM volume would be related to a higher in vivo microglial activation PET binding. However, Malpetti et al. recently reported that anterior temporal microglial activation predicted cognitive decline in MCI and AD (Malpetti et al., 2020). Interestingly, MRI atrophy measures were not predictive over and above the PET signals. Second, and at variance with Femminella et al. (2019), our analyses were performed across the spectrum of MCI to AD, subjected to a corrected significance threshold and, most importantly, accounting for disease severity. Of note, very similar results were obtained both for VBM and SBM using a model not accounting on disease severity. In addition, we used whole-brain voxel/vertex-wise correlational analyses of ^11^C-PK11195 instead of solely assessing its relationship with regional hippocampal volume.

Although our results suggest that increased neuroinflammation is related to cortical thinning and GM atrophy converging in parietal areas, the temporal lobe does not seem to be as severely affected (Fig. 3, Fig. 4). We hypothesize that structural damage, especially in the medial temporal region, is similarly impaired along the spectrum of our milder (MCI) and more severely impaired (AD) participants. The VBM and SBM comparisons between AD and MCI did not show a significant difference in the left medial temporal lobe (Supplementary Fig. 1). Therefore, ^11^C-PK11195 correlational analyses with structural imaging of the temporal lobe would seemingly suffer from a floor effect.

As expected, global cognition as measured with the total ACE-R score was correlated with hippocampal atrophy and parahippocampal cortical thinning (Deweer et al., 1995; Kohler et al., 1998; Ossenkoppele et al., 2019). However, we did not find a significant correlation with ^11^C-PK11195 binding. In fact, our mediation analysis showed that neither a direct nor an indirect effect mediating structural damage was observed for ^11^C-PK11195 in relation to cognitive decline. However, the protective or detrimental role of microglial activation on cognitive deterioration would benefit from longitudinal analyses accounting for more “upstream” pathomechanisms, such as amyloid or tau deposition in addition to neurodegeneration. In fact, recent evidence suggests a relationship between higher microglial activation and cognitive impairment in AD especially when associated with altered functional connectivity (Passamonti et al., 2019).

The present study has limitations. First, our results have been obtained on a relatively modest sample of subjects, so this would require confirmation in larger groups, especially to evaluate MCI and AD patients separately and potentially distinguish the positive or negative effects of microglial activation. Second, the present relationship between microglial activation and structural integrity is based on data obtained with a cross-sectional design. Therefore, longitudinal data are required to fully assess the spatial and temporal interplay between structural imaging and neuroinflammation in AD. Choosing single ROI instead of a composite frontotemporal ROI as we did for ^11^C-PK11195 analyses may have revealed associations in mediation analysis not present with a composite measure, although such multiple analyses would have raised other issues regarding type 1 errors, and missed effects that were distributed over several regions (as captured by the composite score).

The use of ^11^C-PK11195 targeting TSPO cannot represent the full extent of central inflammation which is also determined by other factors than microglial activation. In fact, other targets such as astrocyte activation should be assessed in the future as they might play a role in the neurodegeneration cascade (Acosta et al., 2017; Carter et al., 2019). As a first-generation TSPO ligand, ^11^C-PK11195 has shown lower sensitivity than second-generation tracers (Vivash and O'Brien, 2016). However, it has the advantage of not being significantly affected by the genetic polymorphism (e.g., rs6971 single nucleotide) altering the binding of second-generation ligands, such as ^11^C-PBR28 or ^18^F-DPA-714 (Owen et al., 2012; Varley et al., 2015).

In conclusion, we present new evidence of the in vivo association between microglial activation and gray matter changes in the AD spectrum. Longitudinal studies are required to determine the dynamic relationship between inflammation and structural changes within subjects.

## Disclosure statement

NN, MM, EM, GBW, WRB-J, SFC, LP, TDF, and YTH report no disclosures relevant to the present article. FIA received academic grant support from 10.13039/100006775GE Healthcare, and served as a consultant for Avid and Cantabio, all for matters not related to the current study. JBR has been a consultant for Asceneuron and Syncona, and has received academic grant funding from AZ-10.13039/501100004628MedImmune, Janssen, and 10.13039/100004312Lilly, unrelated to this study. JTO'B received grant support from Avid (10.13039/100004312Lilly), and served as a consultant for Avid and 10.13039/100006775GE Healthcare, all for matters not related to the present study.

## CRediT authorship contribution statement

**Nicolas Nicastro:** Methodology, Investigation, Formal analysis, Writing - original draft. **Maura Malpetti:** Formal analysis, Writing - review & editing. **Elijah Mak:** Formal analysis, Writing - review & editing. **Guy B. Williams:** Methodology, Resources, Writing - review & editing. **W. Richard Bevan-Jones:** Investigation, Writing - review & editing. **Stephen F. Carter:** Methodology, Writing - review & editing. **Luca Passamonti:** Methodology, Investigation, Writing - review & editing. **Tim D. Fryer:** Resources, Writing - review & editing. **Young T. Hong:** Formal analysis, Writing - review & editing. **Franklin I. Aigbirhio:** Supervision, Resources, Conceptualization, Writing - review & editing. **James B. Rowe:** Supervision, Resources, Conceptualization, Project administration, Writing - review & editing. **John T. O'Brien:** Supervision, Resources, Conceptualization, Project administration, Writing - review & editing.
